# Feasibility Study Shows Multicenter, Observational Case-Control Study Is Practicable to Determine Risk of Secondary Breast Cancer in Females With Differentiated Thyroid Carcinoma Given Radioiodine Therapy in Their Childhood or Adolescence; Findings Also Suggest Possible Fertility Impairment in Such Patients

**DOI:** 10.3389/fendo.2020.567385

**Published:** 2020-10-28

**Authors:** Valentina Drozd, Rita Schneider, Tamara Platonova, Galina Panasiuk, Tatjana Leonova, Nataliya Oculevich, Irina Shimanskaja, Irina Vershenya, Tatjana Dedovich, Tatjana Mitjukova, Inge Grelle, Johannes Biko, Christoph Reiners

**Affiliations:** ^1^International Foundation “Arnica,” Minsk, Belarus; ^2^Department of Nuclear Medicine, University Hospital, Würzburg, Germany; ^3^The Center of Thyroid Tumors, Minsk City Oncological Dispensary, Minsk, Belarus; ^4^Center of Medical Rehabilitation and Balneotherapy, Minsk, Belarus; ^5^Clinical Laboratory “MedEx-Lab,” Minsk, Belarus

**Keywords:** thyroid cancer, radiation-induced thyroid cancer, radioiodine therapy, breast cancer risk, infertility

## Abstract

**Objective:** This single-center, observational case-control feasibility study sought to test key elements of a protocol for an eventual long-term international observational case-control study of a larger patient cohort, to evaluate the risk of breast cancer as a second primary malignancy in females with differentiated thyroid cancer (DTC) given radioiodine therapy (RAI) during childhood or adolescence.

**Patients:** Females developing DTC after the Chernobyl accident in Belarus and ≤19 years old at the time of thyroid surgery were enrolled: patients given RAI (*n* = 111) and controls of similar age not given RAI (*n* = 90).

**Results:** One case of breast cancer was newly diagnosed among the RAI patients, but none in controls. Patients given RAI significantly less frequently needed 2^nd^ surgeries than did controls (23%, 26/111 vs. 39%, 35/90, *P* < 0.05); the main indication for such procedures usually is suspicion of local recurrence. RAI patients appeared to have had more frequent reproductive difficulties than did controls: 78% (87/111) of the former vs. 93% (84/90) of the latter had a history of pregnancy (*P* < 0.01), and the mean number of pregnancies was 1.5 ± 1.2 in RAI patients vs. 1.9±1.1 in controls (*P* < 0.05). Most notably, infertility was observed in 23% (26/111) of RAI patients vs. 4% (4/90) of controls (*P* < 0.01). In conclusion, a international observational case-control study on breast cancer after DTC in patients given RAI vs. not given RAI appears to be feasible. Additional research and everyday clinical attention should be devoted to reproductive function after RAI in young females.

## Introduction

Most cases of differentiated thyroid cancer (DTC) in childhood, adolescence, and early adulthood can be successfully treated with surgery, radioiodine [iodine-131 (I-131)] therapy (RAI), and thyroid hormone replacement, resulting in 10-year survival rates of 95% and low recurrence rates of 10–30% (1). However, excellent long-term survival may be restricted by an increased risk for second primary malignancy related to RAI and/or other factors (2, 3). It is well-known that the gastrointestinal tract (salivary glands, stomach, colorectum), the genitourinary tract (kidneys, bladder), and the hematopoietic system (leukocytes) are at risk to develop a second primary malignancy after RAI (2–4). I-131 is concentrated by the sodium-iodide symporter, which is expressed not only in thyrocytes, but in epithelial cells of salivary glands, of the stomach, and of the mammary gland too (5). Thus, the female breast may receive relevant radiation doses of 0.4–0.6 Gy from one course of RAI with 6 GBq (6). We recently reviewed the literature on breast cancer as a second primary malignancy after RAI of DTC with a focus on young patients, and concluded that there is a general association between DTC and breast cancer. The risk for breast cancer after DTC in adults is low—about 2%—and RAI is assumed not to influence that risk substantially, but data in patients given RAI as children and adolescents are sparse. Systematic studies about breast cancer risk in young TC patients after RAI therefore are needed (7, 8).

## Materials and Methods

### Objective and Scope

In addition to the literature review mentioned above, we performed a preliminary multicenter registry survey to evaluate the availability of sufficient patient data for a subsequent international multicenter observational case-control study in children and adolescents with DTC (7, 8). In parallel, to develop a protocol for such a multicenter study, we performed an observational case-control feasibility study in small samples of young female patients with DTC after RAI and of controls with DTC not treated with RAI. Both the survey and the feasibility study were sponsored by the German Federal Office for Radiation Protection (8). The sponsor had no involvement in the content or the decision to publish this paper.

### Patients and Study Design

As part of a variety of collaborative international humanitarian and scientific projects on radiation-induced DTC in Belarussian patients after the Chernobyl nuclear reactor accident (3, 8–15) after thyroid surgery in Minsk (Belarus), children and adolescents with advanced DTC were treated with high-activity RAI in two German academic tertiary referral centers, the University Hospitals of Essen (1992–1993) and Würzburg (1994–2007). Altogether, more than 1,000 cycles of RAI were performed in roughly 250 patients (14) with one or more of pT4 pN1, or pM1 TNM tumor stages. Selection was made without direct influence from the German side.

**Figure 1:**
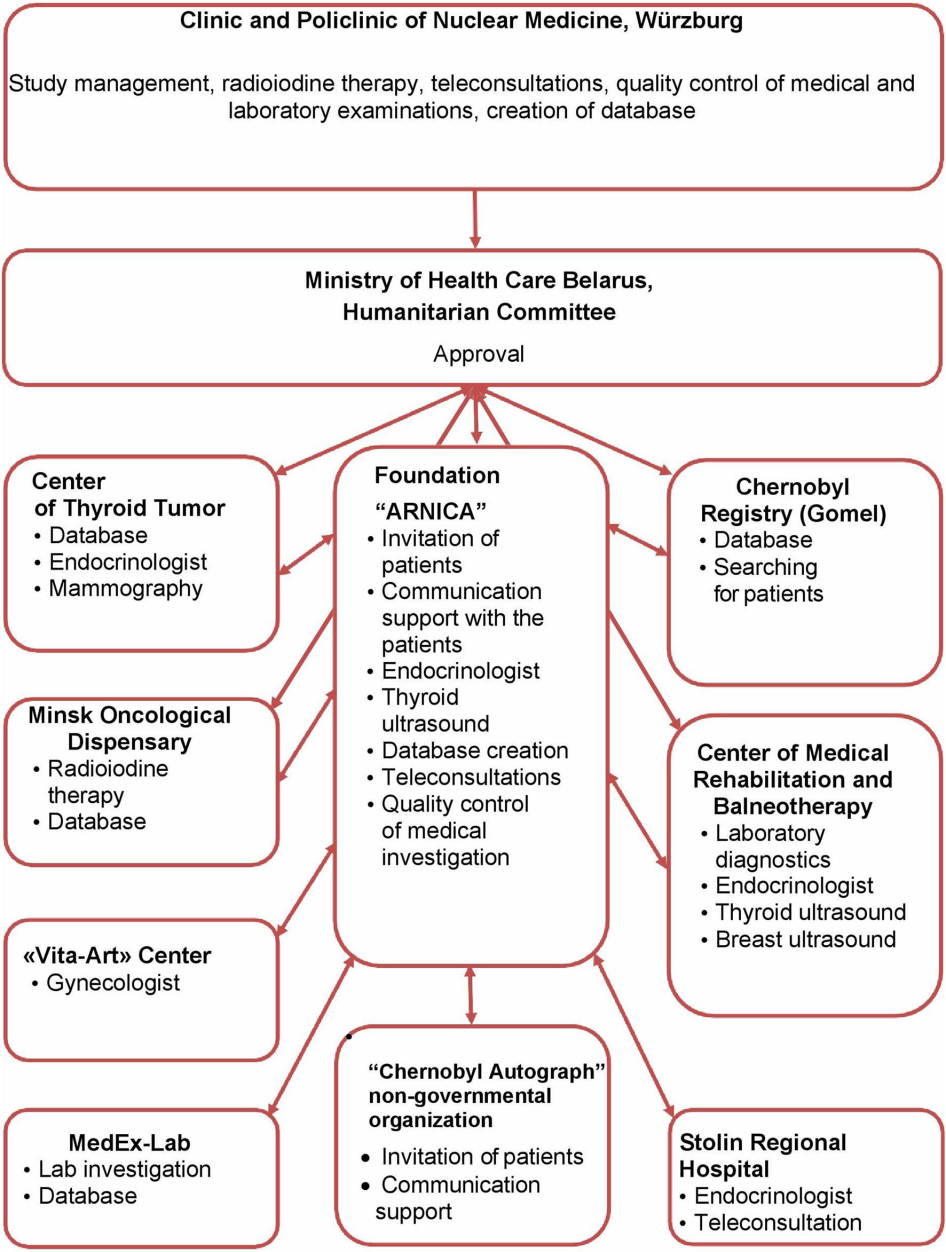
Participating institutions and their tasks. This diagram describes the participation and interaction of various institutions in the implementation of Feasibility Study for determine a risk of secondary breast cancer in females with differentiated thyroid carcinoma given radioiodine therapy in their childhood or adolescence.

With approval of the Ministry of Health and the Humanitarian Committee of Belarus, following treatment in Germany, the patients were followed-up in Minsk by experienced endocrinologists and gynecologists of the Center of Thyroid Tumors, Minsk City Oncological Dispensary, the International Foundation “Arnica,” the “Vita-Art” Health Center, and the “MedEx-Lab” clinical laboratory in cooperation with institutions in Belarus including the Chernobyl Registry, the Center of Medical Rehabilitation and Balneotherapy, the Stolin Regional Hospital, and the “Chernobyl Autograph” non-governmental organization (Figure 1).

We decided to carry out our observational case-control feasibility study in roughly 100 female patients who were age ≤19 years at the time of thyroid surgery during the 1992–2007 treatment period (“RAI patients”). An equal number of patients with DTC of similar age who did not receive RAI would serve as controls. More than 200 potential “RAI patients” group members and ca. 120 potential controls, all females, received invitations for a comprehensive cost-free health check in the outpatient clinic of the “Arnica” Foundation and the “Vita-Art” Health Center. Reimbursement of study participants' travel costs was guaranteed.

### Study Protocol and Quality Assurance

In all participants, the medical and radiological history related to the Chernobyl accident was documented using questionnaires developed for this study. In addition, in the RAI patient group, relevant RAI-related data, e.g., therapeutic activity per course, number of courses, and cumulative activities (in GBq), were registered.

Patients and controls underwent clinical examination including palpation and sonographic examination of the neck and breast. If abnormalities were detected in the breast screening sonogram, then mammography, magnetic resonance imaging, or both followed. The breast cancer screening program used in this study was recommended by the International Late Effects of Childhood Cancer Guideline Harmonization Group (16) for young patients with non-breast cancers who had radiation exposure of the breast. Ultrasonography equipment consisted of a Logiq E” Scanner with 12 MHz-probe (GE Healthcare, Chicago, IL, USA). For quality assurance of breast imaging, findings were documented according to the BI-RADS classification, 5th edition (17).

Study participants also underwent comprehensive biochemical screening based on testing which was carried out at “MedEx-Lab” clinical laboratory in Minsk, with quality control at the laboratory of the Clinic and Polyclinic for Nuclear Medicine in Würzburg, Germany. For classification of remission/progression after therapy of DTC, thyroglobulin and anti-thyroglobulin antibody status were determined. Levothyroxine replacement was checked with determination of thyroid-stimulating hormone (TSH) and free thyroxine (fT4) concentrations. Variables of calcium-phosphate metabolism (parathormone, calcium, and phosphorus levels) were also quantitated. As part of the general health check, blood cell counts, lipid, liver, and pancreas status, and sex hormone status were determined. An automatic immunochemical analyser (COBAS e 411, Roche Diagnostics, Risch-Rotkreuz, Switzerland) was used to measure hormone variables and the COBAS INTEGRA 400 plus automatic analyser (Roche Diagnostics) served for determination of other biochemical variables. The clinical laboratory in Minsk used commercial control sera (Lyphochek® 1, 2, 3; Biorad Company, Hercules, CA, USA) for daily quality assurance checks as well as for external comparisons with the lab of the Clinic and Polyclinic for Nuclear Medicine in Würzburg. Both labs followed the rules of the authorities in Belarus and Germany that govern participation in blinded studies.

### Database, Data Protection, and Patients' Rights

A customized Access database (Microsoft, Redmond, WA, USA) was created for this study. A trained medical documentation assistant entered into this database de-identified data from the records of the study medical and biochemical examinations, which data had been generated by physicians and laboratory staff. The medical documentation assistant regularly made plausibility checks and flagged missing data. Before enrolment, study participants gave their informed consent for participation in the study and were over 18 at the time of data collection and analysis of de-identified data.

### Statistics

Aggregate tabulated results are presented here with arithmetic means ± standard deviations (SDs) and/or medians and minimum–maximum ranges, as appropriate. For intergroup statistical comparisons, Student *t*-test was used for constant variables and chi-square test for frequencies. Results with probability of error <5% were considered to be significant.

## Results

### Study Group

Altogether 111 RAI patients and 90 controls, respectively, were enrolled in the study, which was performed from 2016–2017. Selected characteristics of these groups of study participants are presented in Table 1.

**Table 1:** Characteristics of RAI patients and controls.

**Variable**	**RAI patients *n* = 111**	**Control *n* = 90**	***P***
**Age at thyroid surgery (years)**			
Mean±SD	12.7 ± 3.4	14.3 ± 3.4	<0.002
Median (minimum–maximum)	12.3 (4.2–18.9)	14.7 (6.8–18.9)	
**Extent of thyroid surgery % (*****n*****)**			
Total thyroidectomy	100% (111)	53% (48)	<0.001
Hemithyroidectomy	0 (0.0%)	47% (42)	
**Number of surgeries for DTC % (*****n*****) of subjects in category**			
1	72% (80)	59% (53)	<0.05
2	23% (26)	39% (35)	<0.05
>2	5% (5)	2% (2)	n.s.
**pTNM stage % (*****n*****)**			
T1	43% (48)	90% (81)	<0.001
T2	37% (41)	9% (8)	<0.001
T3	20% (22)	1% (1)	<0.001
N0	9% (10)	60% (54)	<0.001
N1	91% (101)	40% (36)	<0.001
M0	75% (83)	100% (90)	<0.05
M1^*^	25% (28)	0 (0%)	<0.001
**Radioiodine therapy RAI courses (*****n*****)**			
Mean±SD	2.9 + 2.1	–	ND
Median (minimum–maximum)	2 (0–10)	–	ND
**Cumulative RAI activity (GBq)**			
Mean ± SD	10.6 ± 9.5	–	ND
Median (minimum–maximum)	7.4 (2.0–43.0)	–	ND
**Age at enrolment (years)**			
Mean ± SD	33.4 ± 2.6	35.3 ± 3.8	<0.001
Median (minimum–maximum)	32.6 (28.3–42.3)	34.6 (27.2–43.1)	
**Duration of follow-up after thyroid surgery (years)**			
Mean ± SD	20.9 ± 3.9	20.7 ± 4.0	n.s
Median (minimum–maximum)	21.3(13.6–27.3)	21.6(10.5–29.4)	

*Due to rounding, percentages may not add up to 100%*.

*ND, not done; n.s, not statistically significant; RAI, radioiodine (I-131) therapy; SD, standard deviation*.

* *Based on post-operative I-131 whole body scan*.

The RAI patient and control groups differed in certain demographic, disease, and treatment characteristics; most differences were attributable to history of RAI being the key criterion for inclusion into one or the other of the groups. On average, RAI patients were ca. 1.5 years younger at the time of surgery than were the controls (*P* < 0.002). Unsurprisingly, all patients given RAI had undergone total thyroidectomy, because as complete as possible resection of thyroid tissue (whether malignant or healthy) is a precondition for RAI. In contrast, only a bit more than half of controls had undergone such surgery (*P* < 0.001). RAI patients less frequently needed a second surgical intervention (23 vs. 39%, *P* < 0.05). Significantly larger proportions of RAI patients had >pT1 primary tumor, presence of lymph node metastasis, or distant involvement, reflecting disease characteristics likely to have contributed to the decision for or against RAI. In both groups, however >95% of cases were histologically classified as papillary cancers.

Regarding Chernobyl-related radiation exposure, there were no inter-group differences in residence on contaminated ground, consumption of contaminated food and milk, or use of iodine thyroid blocking around the time of the accident, the last of which was reported in <5% of members of either group (data not shown). The respective percentages of family members of RAI patients or controls with thyroid cancer (7 vs. 4%), breast cancer (6 vs. 8%) or any type of cancer (30 vs. 29%) showed no statistically significant differences. Additionally, there were no differences in BMI, age at menarche, regularity of the menstrual cycle, prevalence of diabetes, alcohol abuse, or use of oral contraceptives.

Concerning reproductive history, 78% of RAI patients (87/111) and 93% of controls (84/90) reported history of pregnancy, a statistically significant difference (*P* < 0.01). The mean numbers of pregnancies also differed between these groups (1.5 ± 1.2 vs. 1.9 ± 1.1; *P* < 0.05). In the RAI group, 50% of mothers (44/87) had practiced breast-feeding, in the control group, 53% (45/84). Remarkably, infertility according to the World Health Organization definition, i.e., inability to become pregnant despite regular sexual intercourse without contraception, was observed in 23% of RAI patients (26/111). Two of these 26 women were treated for infertility for a long time and then were able to give birth to healthy children using *in vitro* fertilization. Only 4% of controls (4/90) (*P* < 0.01) were treated for infertility and in this group, 2 women were not married.

### Current Physical and Biochemical Findings

Enlarged neck lymph nodes, reflecting possible DTC recurrence, were detected by ultrasound in 14% of the RAI group (16/111) and 27% in the control group (24/90) (*P* < 0.05). DTC recurrences were found sonographically and subsequently histologically verified by fine needle aspiration biopsy in 2 control patients (2%), but no RAI patient.

Presumably due to their having completely-excised thyroid glands, RAI patients needed higher mean thyroid hormone replacement doses of levothyroxine than did controls, 2.3 ± 0.5 μg/kg body weight vs. 2.0 ± 0.9 μg/kg body weight (*P* < 0.001). However, mean fT4 values did not differ between the groups, nor did mean TSH concentrations (Table 2).

**Table 2:** Current biochemical findings by category, % (*n*).

**Variable (relationship to reference range)**	**Reference range**	**RAI patients *n* = 111**	**Controls n = 90**	***P*-value**
**TSH (mIU/L)**				
≤ 0.10 (below)	0.27–4.2	45% (50)	40%(36)	n.s.
>0.10–0.27 (within)		12% (13)	11%(10)	n.s.
>0.27–4.2 (within)		36% (40)	44% (40)	n.s.
>4.2 (above)		7% (8)	4% (4)	n.s.
**fT4 (pmol/L)**				
<12 (below)	12–22	1% (1)	3% (3)	n.s.
12–22 (within)		55% (61)	62% (56)	n.s.
>22 (above)		44% (49)	34% (31)	n.s.
**Tg (ng/ml)**				
<1.0 (within)	<1^*^	93% (103)	54% (49)	<0.001
≥1.0 (above)		7% (8)	46% (41)	<0.001
**PTH (ng/mL)**				
<15 (below)	15–65	35% (39)	9% (8)	<0.001
15–65 (within)		64% (71)	87% (78)	<0.001
>65 (above)		1% (1)	4% (4)	n.s.

*Due to rounding, percentages may not add up to 100%*.

*fT4, free thyroxine; n.s, not significant; PTH, parathormone; Tg, thyroglobulin; TSH, thyroid-stimulating hormone*.

* *After thyroidectomy*.

Additionally, thyroglobulin levels were below the level of detectability (1 pg/mL) in a significantly greater proportion of RAI patients (*P* < 0.001). Presumably due to RAI patients' more frequent radical operations, decreased parathormone levels (<15 pg/mL) signaling postoperative hypoparathyroidism, a well-known complication of thyroid surgery, were much more frequent in that group than in the control group (*P* < 0.001). Corresponding to a greater frequency of decreased parathormone levels after thyroidectomy and RAI, blood calcium was decreased in 36% of RAI patients (40/111) and 21% of controls (19/90) (*P* < 0.05), while phosphate was increased in 28% (32/111) and 9% (8/90), respectively (*P* < 0.002).

Neither RAI patients nor controls differed, or showed significant abnormalities in blood cell counts (data not shown). Concerning blood lipid values, however, while the groups did not differ, they both had a high prevalence of abnormalities. Total cholesterol was above the reference range in 47% of RAI patients (52/111) and 43% of controls (39/90), while low-density lipoprotein was above the reference range in 73% of RAI patients (81/111) and 72% of controls (65/90). High-density lipoprotein levels were below the reference range in 43% and 44% of these groups, respectively. No abnormalities were seen in the liver enzymes alanine aminotransferase and or aspartate aminotransferase. Elevated blood glucose levels were found in 5% of RAI patients (6/111) and 8% of controls (7/90). Neither the differences in liver enzymes, nor those in blood glucose were significant.

Regarding the sex hormones, there were no significant inter-group differences in luteinizing hormone, follicle-stimulating hormone, anti-Müllerian hormone, prolactin, estrogen, or testosterone concentrations (data not shown). Progesterone levels were less frequently abnormally decreased in RAI patients compared to controls (16%, 18/111 vs. 41%, 41/90; *P* < 0.01).

### Breast-Related Findings

Previous events of clinical relevance to breast cancer such as breast trauma, lumps, local pain, or mastitis were only rarely reported, in one to three patients, with no differences between groups. Similarly, findings like skin abnormalities, conspicuous or secreting mammillae, and palpable indurations or lumps on inspection and palpation were rare and evenly distributed among RAI patients vs. controls.

Table 3 summarizes the results of breast sonography. Sixty-one percent of RAI patients (68/111) and 68% of controls (61/90) had completely normal findings. This difference was not statistically significant; nor were the differences regarding frequencies of diffuse changes, focal lesions, cysts, fibromas, or detectable lymph nodes. Ninety-six percent of sonograms in patients given RAI and of 100% in controls were classified into non-suspicious BI-RADS categories.

**Table 3:** Breast ultrasonography findings.

**Variable**	**RAI patients *n* = 111**	**Controls *n* = 90**	***P***
**Normal findings, % (*****n*****)**			
Yes	61% (68)	68% (61)	n.s.
No	39% (43)	32% (29)	
**Diffuse changes, % (*****n*****)**			
Yes	26% (29)	22% (20)	n.s.
No	74% (82)	78% (70)	
**Focal lesions, % (*****n*****)**			
Yes	11% (13)	9% (8)	n.s.
No	88% (98)	91% (82)	
Largest focal lesion (mm), mean ± SD	8.1 ± 4.5	7.5 ± 3.1	n.s.
Cysts, %(*n*)	5% (5)	3% (3)	n.s.
Fibroma, %) (*n*)	4% (4)	0% (0)	n.s.
**Lymph nodes, % (*****n*****)**			
None	97% (108)	99% (89)	n.s.
Single	3% (3)	1% (1)	n.s.
Multiple	0% (0)	0% (0)	
**Localization of lymph nodes, % (*****n*****)**			
Axillary	3% (3)	1% (1)	n.s.
Other	0% (0)	0% (0)	
**BI-RADS category**, ***n*** **(%)**			
Category 0	0% (0)	0% (0)	n.s.
Category 1	61% (68)	68% (61)	n.s.
Category 2	35% (39)	32% (29)	n.s.
Category 3	4% (4)	0% (0)	n.s.
Category 4	0% (0)	0% (0)	n.s.
Category 5	0% (0)	0% (0)	n.s.

*Due to rounding, percentages may not add up to 100%*.

*BI-RADS, breast imaging–reporting and data system; n.s, not statistically significant; RAI, radioiodine (I-131) treatment*.

In the 4 RAI patients with BI-RADS category 3 findings, the suspicious lesions were removed surgically, with a histological diagnosis of fibroma in 3 cases and carcinoma in 1.

## Discussion

The aim of this observational case-control feasibility study in two small samples (*n* = ca. 100 each) of young female patients of similar age, whose treatment for DTC either included or did not include RAI, was to develop a protocol for a larger multicenter study with a sufficiently large sample to reliably test the hypothesis “RAI does not increase the risk for breast cancer compared to DTC patients without RAI.” Based on our previously-mentioned literature review and international registry survey, we assumed this risk to be ~ 2.5% and concluded that at least 4,340 patients and 660 controls would be needed to test the hypothesis with adequate statistical power, 80% (7).

This feasibility study delivered a number of expected results. First, concerning numbers of subjects in both groups, it was not surprising that it was more difficult in our patient population to recruit controls (*n* = 90) than patients given RAI (*n* = 111). Most children and adolescents with DTC after the Chernobyl accident presented with relatively advanced tumor stages, in which RAI is indicated. This can be deduced from the observation that 100% of the RAI had (near-) total thyroidectomy, 57% were classified postoperatively as tumor stage >pT1, and 91% as pN1 and 40% as pM1 stages.

Second, an imaging protocol following the recommendations (16) to use the BI-RADS classification (17) in young patients with primary malignancy of non-breast tissues, but radiation exposure of the breast, proved to be feasible. Four cases of BI-RADS category 3 findings were detected among the RAI patients and histologically verified, among them 1 case of breast cancer. This single case, and the lack of cases among the controls, do not allow any statistical conclusions to be drawn regarding breast cancer risk associated with RAI. However, the breast cancer case provides additional evidence for the still-unproven assumption that this tumor is in general rare in young females with DTC (7). On the other hand, at least 3 cases of SPM other than breast cancer, all cancer of the cervix uteri, which were not in the focus of this study, were found by history in 111 RAI patients. These observations align to some extent with data published by Fridman et al. (18) demonstrating that the prevalence of SPM in 4,237 patients treated in Belarus for post-Chernobyl papillary DTC between 1990–2015 was 1% after a 15-year follow-up. Among the 41 patients who developed SPM, hematological malignancies (9 cases), cervical cancer (7 cases), breast cancer (4 cases), and colon carcinoma (4 cases) were most frequently observed (18).

Third, quality checks suggest that out study had reliable biochemistry results. Thyroglobulin levels in blood should be undetectable if no healthy or cancerous thyroid tissue, i.e., no local residues, involved lymph nodes, or distant metastases remain after therapy. The finding that thyroglobulin levels were statistically more frequently measurable in controls than in RAI patients corresponds conversely to the more complete surgical removal of thyroid tissue, and to additional destruction of such tissue by RAI.

According to the current American Thyroid Association guidelines (19), it seems that too high a percentage−40%—of our controls had complete TSH suppression. On the other hand, the corresponding frequency of complete suppression, 45%, in our higher-risk RAI patient group seems to have been too low. There is a debate about TSH target values in the literature, addressing potential cardiovascular complications of complete TSH suppression in low-risk patients (19, 20) such as, e.g., our control group. Additionally, chronic levothyroxine overdosage is suspected of increasing breast cancer risk independent of DTC (21). Reasons for the possibly suboptimal degree of TSH suppression in many of our patients may include the supply situation of levothyroxine necessitating that patients frequently switch among different thyroid hormone preparations. The explanation also may include laboratory monitoring at irregular intervals, since levothyroxine regimens in athyroid individuals must be titrated and re-titrated over time, according to circulating TSH levels.

Not completely unexpected was the appreciable prevalence of laboratory constellations of hypoparathyroidism, i.e., increased circulating phosphate, decreased parathormone, and calcium. However, mostly due to complications of the generally more radical surgery in the RAI patients, the percentage of below-normal parathormone blood levels, 35%, in that group was relatively high, compared to the 9% in controls. Obviously, medication for substitution therapy of hypoparathyroidism needs specific attention in patients treated for DTC at a young age, especially those undergoing total thyroidectomy. Increased levels of serum cholesterol in roughly 45% of patients and controls seem surprising. However, in a recent narrative review of studies focusing on this issue, Bianco and Taylor (22) stressed that a high percentage of patients on levothyroxine replacement after thyroidectomy who have normal TSH levels have elevated cholesterol, and that indeed, >50% of such patients need statins.

This study also had two interesting and particularly noteworthy findings. First, RAI patients significantly less frequently needed 2 or more surgical interventions than did controls (23% vs. 39%, *P* < 0.05). The main indication for 2^nd^ intervention is usually suspicion of local recurrence; notably, ca. 20 of the approximately 120 subjects who were approached to participate as controls had to be excluded because they already had been diagnosed with a DTC recurrence.

Second, only 78% of the post-RAI patients, compared to 93% of controls, reported a history of pregnancy, and the mean numbers of pregnancies differed significantly between groups (1.5 ± 1.2 vs. 1.9 ± 1.1, *P* > 0.05). Remarkably, infertility defined according to recent international standards (23) as inability to become pregnant despite regular sexual intercourse without contraception, was observed in 23% of post-RAI patients, vs. only 4% of controls (*P* < 0.01). It should be noted that Chart of European Health Information Gateway (WHO) for the Belarus demonstrated that fertility rate in 2016 (time of practical implementation of study) was 1.7 births per woman (24). About each fifth couple in Belarus faces infertility, and male infertility accounts for 50% of infertile couples (25).

The literature contains high-level evidence of transient testicular dysfunction in males after RAI, but only very low-level evidence, and conflicting findings, regarding female reproductive outcomes (2, 6). For example, a study by Nies et al. (26) demonstrated that female survivors of DTC who received 131-I treatment during childhood did not appear to have major abnormalities in reproductive characteristics nor in predictors of ovarian failure (26). Giusti et al. (27) found no difference in anti-Müllerian hormone levels in two relatively small samples of patients with DTC undergoing or not undergoing RAI, and that infertility must be considered a low risk, but concluded that 1 out of 2 women with DTC suffer from menstrual dysregulation independent of RAI (27). Yaish et al. (28), however, observed a significant decrease of anti-Müllerian hormone levels 3 months after RAI, with only partial recovery after 12 months. The Yaish et al. study had some limitations, however, because the cohort was inhomogeneous, including patients with DTC or Graves' disease. Further, no control group with similar fluctuations of TSH levels, but not given RAI, was studied (28). Taken together with the limitations of the literature, our observations regarding reproductive history suggest the need for additional, systematic study of the associations of DTC, RAI, thyroid hormone replacement therapy, and gynecological comorbidities, e.g., cervical cancer as an SPM, with female fertility.

Our study has some strengths, and some limitations too. In the former category, the investigation was carried out by experienced clinicians following a strict predefined and quality-assured protocol. Additionally, patients and controls matched well-regarding history of Chernobyl-related radiation exposure, family history of DTC, breast cancer, or other types of cancer, and frequency of potentially confounding breast cancer risk factors, e.g., high BMI, diabetes, alcohol abuse, older age at menarche, irregular menstrual cycle, and use of oral contraceptives. Study groups were relatively small in absolute terms, but large for a single center following patients with childhood DTC. Moreover, follow-up times, ranging between 10–30 years (mean 20 ± 4.0 years) after thyroid surgery, were long. The study results provided assurance that the follow-up protocol properly had among its main focuses the identification of recurrences, optimization of levothyroxine dosing, and replacement therapy for hypoparathyroidism. A weakness of this study is that all participants had been exposed to radiation from the Chernobyl reactor accident, which influences the risk and possibly the clinical behavior of DTC notably (29) and questionably the risk of BC too (30).

In conclusion, a sufficiently-powered subsequent international, multicenter, observational case-control study on breast cancer risk in female patients with DTC given or not given RAI in childhood or adolescence appears to be feasible; key elements of the required protocol for that study, including necessary quality controls, were successfully tested in such patients. Based on the findings of the present study and the results of a corresponding comprehensive literature review (7), the risk for breast cancer in DTC survivors seems not to be high. However, the risk in DTC subtypes, the influence of age (taking into account exposure to RAI around puberty), genetic predisposition for BC by familial history and genotyping as well as genome wide association studies of DTC should be addressed more in detail and additional attention should be paid to reproductive function after RAI. It may be argued that more aggressive DTC, deserving high activities of RAI, could occur in patients with genetic predisposition to second primary cancer. According to a recent review on genome wide association studies a gradual increase in the general risk for differentiated thyroid cancer can be demonstrated with the strongest SNPs but the overall prediction ability appears to be very limited. Up to now, the clinical aggressiveness of DTC and its risk for SPM's by “genomic profiling” is not possible (31).

## Data Availability Statement

All datasets generated for this study are included in the article/supplementary material.

## Ethics Statement

The studies involving human participants were reviewed and approved by Ministry of Health of Belarus. The patients/participants provided their written informed consent to participate in this study.

## Author Contributions

VD: concept development, study management, patients' examination in Minsk, analysis of thyroid cancer database, writing and editing manuscript. RS: study management, management and analysis of thyroid cancer database in Minsk, language editing. TP: database management and analysis of thyroid cancer database in Minsk, statistical data processing. GP: patients' examination in Gomel, analysis of thyroid cancer database in Gomel. TL, NO, and IS: patients' examination in Minsk, analysis of thyroid cancer database in Minsk. IV: laboratory tests, quality control, analysis of thyroid cancer database in Minsk. TD: study management and analysis of thyroid cancer database in Minsk, statistical data processing. TM: laboratory tests, analysis of thyroid cancer database in Minsk. IG: quality control of laboratory tests, analysis of thyroid cancer database in Minsk. JB: concept development, study management, patients' examination in Minsk and in Würzburg. CR: concept development, study management, patient's examination in Minsk and in Würzburg, analysis of thyroid cancer database, writing and editing manuscript. All authors critical review and approval of final version of manuscript.

## Conflict of Interest

The authors declare that the research was conducted in the absence of any commercial or financial relationships that could be construed as a potential conflict of interest.
